# RETRACTION: Vitamin D and Wound Healing: Assessing Skin Barrier Function and Implications for Chloasma Treatment

**DOI:** 10.1111/iwj.70481

**Published:** 2025-04-02

## Abstract

**RETRACTION:** ChenQ. , LiuL. , and ZhangY. , “Vitamin D and Wound Healing: Assessing Skin Barrier Function and Implications for Chloasma Treatment,” International Wound Journal 21, no. 1 (2024): e14541, 10.1111/iwj.14541.38272820 PMC10789544

The above article, published online on 15 January 2024, in Wiley Online Library (http://onlinelibrary.wiley.com/) and its correction (https://doi.org/10.1111/iwj.14893), has been retracted by agreement between the journal Editor in Chief, Professor Keith Harding; and John Wiley & Sons Ltd. Following an investigation by the publisher, all parties have concluded that this article was accepted solely on the basis of a compromised peer review process. The editors have therefore decided to retract the article. The authors did not respond to our notice regarding the retraction.

**RETRACTION:** Q. Chen , L. Liu , and Y. Zhang , “Vitamin D and Wound Healing: Assessing Skin Barrier Function and Implications for Chloasma Treatment,” International Wound Journal 21, no. 1 (2024): e14541, 10.1111/iwj.14541.38272820 PMC10789544

The above article, published online on 15 January 2024, in Wiley Online Library (http://onlinelibrary.wiley.com/) and its correction (https://doi.org/10.1111/iwj.14893), has been retracted by agreement between the journal Editor in Chief, Professor Keith Harding; and John Wiley & Sons Ltd. Following an investigation by the publisher, all parties have concluded that this article was accepted solely on the basis of a compromised peer review process. The editors have therefore decided to retract the article. The authors did not respond to our notice regarding the retraction.

